# Built Environment-Modulated Epigenetics: The Epigenetic Consequences of Architecturally Mediated Allostatic Overload in the Built Environment

**DOI:** 10.3390/ijerph23060688

**Published:** 2026-05-22

**Authors:** Cleo Valentine, Heather Mitcheltree, Isabelle Sjövall, Mohamed Hesham Khalil

**Affiliations:** 1 Department of Architecture, University of Cambridge, Cambridge CB2 1PX, UK; 2 Department of Environmental Health, T.H. Chan School of Public Health, Harvard University, Boston, MA 02115, USA; 3 Division of the Built Environment, RISE Research Institute of Sweden, 114 28 Stockholm, Sweden; 4 Institute of Behavioural Neuroscience, University College London, London WC1E 6BT, UK

**Keywords:** built environment-modulated epigenetics, allostatic load, built environment design, chronic stress, DNA methylation, neuroarchitecture, transgenerational inheritance

## Abstract

**Highlights:**

**Public health relevance—How does this work relate to a public health issue?**

- The built environment is a near-continuous, population-wide exposure, yet its potential role as a chronic stressor capable of biologically embedding health risk has been largely overlooked in environmental epigenetics, despite robust evidence that chemical pollutants, air pollution, and psychosocial stressors produce lasting epigenetic modifications.
- Stress-inducing architectural features (e.g., spatial enclosure, low ceilings, visually discordant facades, circadian-disrupting lighting) are associated with stress responses and may sustain activation of the HPA and SAM axes, the same pathways through which other environmental stressors are known to drive maladaptive DNA methylation and histone modifications relevant to inflammation, neuroendocrine regulation, and disease susceptibility.

**Public health significance—Why is this work of significance to public health?**

- The built environment-modulated epigenetics (BEME) framework proposes that chronic exposure to stress-inducing built environments may contribute to durable epigenetic changes with potential transgenerational consequences, meaning design decisions could shape health trajectories not only of current occupants but of future generations.
- Because adverse built environment exposures are unevenly distributed across socioeconomically disadvantaged communities, BEME offers a mechanistic lens for understanding how spatial inequities may become biologically embedded, while also identifying enriched and biophilic design features (e.g., green space, daylight, affordances for physical activity) as potential protective levers for resilience and health equity.

**Public health implications—What are the key implications or messages for practitioners, policy makers and/or researchers in public health?**

- For researchers: A phased empirical agenda is needed, including animal model proof-ofconcept studies, longitudinal human cohorts spanning sensitive developmentalwindows, and intervention trials, supported by a standardised panel of endocrine, inflammatory, and epigenetic biomarkers (e.g., cortisol, IL-6, *NR3C1*/*FKBP5*/*BDNF* methylation, epigenetic ageing clocks) to test BEME and quantify exposure–response relationships.
- For practitioners and policy makers: Architectural design, urban planning, and housing policy should be recognised as potential determinants of long-term biological programming, warranting precautionary, evidence-aware design standards that minimise chronic architectural stressors and prioritise restorative, enriched environments, particularly in settings serving children, pregnant individuals, and populations facing cumulative environmental disadvantage.

**Abstract:**

The concept of architecturally mediated allostatic overload has established that chronic exposure to stress-inducing built environments can elicit stress responses within the body, overwhelming regulatory systems and contributing to adverse health outcomes through sustained activation of stress response pathways. Recent advances in epigenetics, combined with emerging evidence of environmental stress-induced epigenetic modifications, suggest that the health impacts of chronic built environment stress may extend far beyond previously understood physiological consequences. This paper introduces the theoretical concept of “built environment-modulated epigenetics” (BEME), extending the framework of architecturally mediated allostatic overload to consider how chronic exposure to stress-inducing built environments may create lasting epigenetic modifications with potential transgenerational implications. We propose that prolonged activation of the hypothalamic–pituitary–adrenal (HPA) and sympathetic-adreno-medullary (SAM) axes by built environment stressors may result in maladaptive DNA methylation and histone modifications affecting stress-responsive genes, similar to documented effects of environmental toxins, air pollution, and psychosocial stressors. Given robust evidence that environmental stressors can create transgenerational epigenetic effects, this theoretical framework suggests that stress-inducing built environments may impact not only current occupants, but future generations through heritable epigenetic modifications. This extension of architecturally mediated allostatic overload theory fundamentally challenges traditional approaches to architectural design and urban planning, positioning the built environment as a potential determinant of long-term epigenetic programming.

## 1. Introduction

Epigenetics has transformed environmental health research by demonstrating that lived exposures can become biologically embedded via regulatory changes in gene expression, without altering the underlying DNA sequence [1,2]. An expanding field of research has demonstrated that exposure to environmental factors such as chemical pollutants, airborne particulates, and chronic psychosocial stress are associated with epigenetic variation in pathways implicated in inflammation, neuroendocrine regulation, and disease susceptibility [3,4,5,6]. These associations are often long-lived and may be particularly significant when exposure occurs during developmentally sensitive windows [2,3]. However, claims of transgenerational inheritance in humans require particular caution, as intergenerational resemblance can arise through shared context, prenatal mechanisms, or ongoing exposure rather than germline transmission [2]. Within this broader evidence base, chronic stress has been linked to epigenetic regulation of HPA axis function and neurotrophic signalling, with potential implications for enduring shifts in stress reactivity and health risk trajectories [4].

Parallel developments in neuroarchitecture have established that built environments can function as chronic environmental stressors, triggering neurophysiological stress responses through sustained activation of the HPA and sympathetic-adreno-medullary (SAM) axes. The framework of architecturally mediated allostatic overload demonstrates that specific built environment features, including the degree of spatial enclosure, visual patterns, lighting conditions, and geometric forms, can induce stress responses and have implications for chronic disease risk [7,8]. However, while environmental epigenetics has extensively documented how environmental exposure to chemicals, air pollution, and psychosocial stressors create lasting biological changes, the design of the built environment has been largely overlooked as a potential epigenetic modulator. This represents a critical gap in our understanding of the impact of the design of the built environment and sustained exposure to architectural stressors.

This paper introduces the concept of “built environment-modulated epigenetics” (BEME) as a theoretical framework. We propose that the same mechanisms by which environmental toxins and psychosocial stressors induce epigenetic modifications may operate in response to chronic exposure to architectural stressors. This hypothesis rests on the convergence of four well-established research findings:

1. Architectural environments trigger chronic stress responses through sustained activation of HPA and SAM axes.
2. Chronic environmental stressors create lasting maladaptive epigenetic changes in stress-responsive genes through DNA methylation and histone modifications [9].
3. Chronic stress is often associated with alterations to gene expressions where vital genes related to brain structure and immune system are downregulated [10,11,12].
4. Maladaptive epigenetic modifications can be transmitted across generations, often affecting the un-exposed generation more than the directly exposed individual [13].

This paper theorises that when taken together, these findings suggest that built environments that act as architectural stressors may function as environmental inputs capable of engaging biological stress-regulation pathways, with the potential to contribute to durable epigenetic modifications under sustained exposure conditions. If substantiated empirically, such mechanisms would imply that the health impact of built environment exposure extends beyond acute or short-term physiological effects, and may result in longer-term epigenetic modifications.

## 2. Environmental Epigenetics and Chronic Stress

### 2.1. Mechanisms of Environmental Epigenetic Programming

Environmental epigenetics has established that external factors, such as toxicants, can induce lasting changes in gene expression through molecular modifications that do not alter the underlying DNA sequence but potentially lead to the inheritance of maladaptive changes [14,15,16]. These modifications primarily involve DNA methylation, a normal modulatory process defined as the addition of methyl groups to a cytosine base in DNA [17], which becomes maladaptive if it becomes persistent or stress-associated. Histone modifications, including acetylation, methylation, and phosphorylation of histone proteins around which DNA is wrapped, play a key role in regulating gene expression [18,19]. These chemical modifications function as regulatory signals that determine which genes are actively expressed in different cell types.

DNA methylation is a covalent chemical modification in which a methyl group is added to a cytosine residue [17]. This modification is generally associated with gene silencing (transcriptional repression), as methylated DNA can hinder the binding of transcription factors and recruit proteins that promote chromatin compaction. Because DNA methylation patterns are relatively stable, they can provide long-term regulation of gene activity and in many instances can result in intergenerational transmission of altered gene expression [20]. Altered methylation patterns can disrupt normal gene expression, influencing developmental trajectories, metabolic regulation, immune responses, and susceptibility to various health conditions. In this way, DNA methylation serves as a key epigenetic mechanism linking environmental factors and context-dependent life experiences to long-term changes in gene function [21].

Histone modifications provide more dynamic regulatory mechanisms, either activating or repressing gene transcription depending on the specific modification and genomic location [18,22,23]. DNA is wrapped around histone proteins to form a structure called chromatin [24,25]. These histones can undergo small chemical changes, known as post-translational modifications, including acetylation, methylation, and phosphorylation [26,27]. Such modifications mainly occur on the histone tails and can change how tightly or loosely DNA is packaged. Acetylation usually makes chromatin more open and active, methylation can either activate or repress genes depending on the location, whereas phosphorylation impacts cell division and DNA repair. Together, these changes form a “histone code” that helps control which genes are active in a cell, influencing development, cell function, and disease [28,29,30,31].

The key insight from environmental epigenetics research is that these modifications respond to environmental cues, creating a ‘molecular memory’ of environmental stimuli or experiences that can influence gene expression long after the initial environmental stimulus is removed [32]. This mechanism enables the environment to create biological changes that persist beyond the duration of the specific exposure, changes that can have intergenerational ramifications.

### 2.2. Environmental Stressors and Epigenetic Modifications

Chemical stressors, including endocrine disruptors, polycyclic aromatic hydrocarbons, and heavy metals, induce specific patterns of maladaptive DNA methylation and histone modifications [33]. Air pollution exposure, particularly fine particulate matter, has been associated with epigenetic ageing and altered methylation patterns in genes regulating inflammation and oxidative stress responses [34,35]. Similarly, psychosocial stressors have emerged as particularly potent epigenetic modulators [36]. Studies of childhood adversity, trauma, and chronic stress have revealed lasting epigenetic modifications in stress-responsive genes, particularly those regulating HPA axis function [4]. Notably, these stress-induced epigenetic changes often persist long after the initial stressor is removed, creating biological modifications that influence stress reactivity and health outcomes throughout an individual’s life [13].

The literature relating to environmental epigenetics emphasises several critical factors that influence epigenetic programming: dose (intensity of exposure) [37], composition (specific characteristics of the stressor) [38], and window of exposure (developmental timing) [39]. These factors determine both the magnitude and persistence of epigenetic modifications, with early-life exposures often creating the most profound and lasting effects. The critical windows of enhanced sensitivity to epigenetic modifications are particularly evident during periods of rapid development and cellular differentiation [40]. Prenatal and early postnatal periods represent windows of heightened epigenetic plasticity during which environmental influences can create lasting changes in gene expression patterns that persist throughout life [39]. These developmental considerations are particularly relevant to BEME, and can have implications for exposure to different built environment characteristics at critical developmental periods.

However, rather than functioning solely as mechanisms of risk, epigenetic processes may also support adaptive responses that protect against environmental stressors. Longitudinal genetic studies demonstrate that developmental trajectories are shaped not only by inherited predispositions but also by the dynamic interplay between genes and environments [41]. For instance, exposure to environmental enrichment provides the brain with stress resilience ability during exposure to stress [42]. Exposure to an enriched environment, encompassing social interaction, physical activity, and novelty, has been shown to reverse biological effects associated with prenatal stress, including those transmitted across generations [43]. These reversals operate at both the behavioural and epigenetic level, the latter referring to changes in gene expression that occur without alteration of the underlying DNA sequence. McCreary and Metz [44] demonstrated that environmental enrichment increased histone acetylation at the BDNF gene in adulthood, a process that renders the gene more transcriptionally active. Given BDNF’s critical role in neuroplasticity, learning, and mood regulation, its upregulation represents a significant neurobiological outcome. These findings suggest that positive environmental experience in adulthood may partially attenuate the enduring molecular consequences of early or intergenerational stress. Recent work further highlights the role of individuals as active co-creators of their environments, shaping exposures in ways that can reinforce resilience [45]. Within built environment contexts, features such as access to green spaces, daylight, or opportunities for physical activity and social connection may therefore act as protective environmental inputs, supporting epigenetic profiles that mitigate stress and promote long-term health [46]. This perspective suggests that enriched built environments could potentially represent critical leverage points for fostering resilience. This hypothesis has led to the establishment of environmental enrichment frameworks for humans, explaining how environmental affordances for physical activity can increase BDNF in humans [47], and how architecturally mediated Neurobiophilia can restore the connection with nature to increase BDNF and support hippocampal neurogenesis [48]. Combining both frameworks is modelled as a predictor for both stress recovery and long-term stress resilience [49], suggesting that the built environment has a bidirectional relationship with stress and epigenetics as a result of architectural enrichment and built environment stressors.

Exposure to the built environment is distinctive in its chronicity: architectural and urban conditions constitute near-continuous background exposures rather than discrete or episodic stressors. Emerging evidence from environmental epigenetics suggests that urban contexts are associated with differential DNA methylation across gene networks implicated in stress regulation and inflammation. For example, Galea et al. [50] propose that DNA methylation may be one mechanism through which urban environmental factors contribute to mental disorders. In a Detroit-based study, Galea et al. [50] reported that methylation profiles distinguish individuals with and without depression/PTSD, and reviewed evidence linking neighbourhood disadvantage and segregation to mental health. While the definition of the built environment does not isolate architectural features per se, it indicates that aspects of the built environment are biologically relevant at the molecular level.

Separately, substantial literature demonstrates that chronic psychosocial stress is associated with epigenetic variation in stress-responsive pathways, including genes involved in hypothalamic–pituitary–adrenal (HPA) axis regulation and neuroplasticity (e.g., NR3C1, FKBP5, TH, BDNF) [4,51]. Such associations are more consistently observed under conditions of sustained or repeated exposure, as opposed to acute stressors, suggesting that duration and persistence are critical determinants of epigenetic embedding.

Importantly, exposure to stress-inducing environmental conditions is unevenly distributed across populations. Research on environmental riskscapes shows that socioeconomically disadvantaged communities are more likely to experience chronic exposure to adverse built environments, alongside other stressors [52]. Studies of neighbourhood disadvantage have identified systematic differences in DNA methylation profiles related to inflammatory and cardiovascular risk [53], raising the possibility that unequal environmental exposure profiles contribute to the biological embedding of health inequalities.

Access to green space may provide compelling evidence that environmental features can create measurable epigenetic changes across populations [54]. For instance, recent longitudinal analyses show that sustained exposure to ‘nature’ in early adulthood is associated with slower epigenetic ageing, even after accounting for demographic, lifestyle, and neighbourhood factors [55]. Similarly, maternal residential green space exposure is positively associated with increased DNA methylation of the serotonin receptor gene HTR2A in placental tissue [56]. Specifically, for each interquartile range increment in total green space, HTR2A methylation increased by 1.47%, 1.52%, and 1.42% within 1000 m, 2000 m, and 3000 m buffers around the maternal residence, respectively [56]. Furthermore, residences with surrounding nature within 50 m and 100 m buffers showed 3.00% and 1.98% higher HTR2A methylation, respectively, compared to those without nearby nature [56]. These findings suggest that prenatal green space exposure may epigenetically modulate serotonergic signalling pathways in the placenta, with potential downstream consequences for foetal neurodevelopment. Framing BEME within this perspective highlights the potential of design interventions to promote better health equity through access to restorative environmental features.

Despite extensive documentation of epigenetic responses to chemical environmental stressors and psychosocial stress, the potential for built environments to create similar modifications remains largely unexplored. If built environment stressors induce similar transgenerational epigenetic changes, the health impacts of built environment design decisions may extend far beyond the initially exposed population, creating biological legacies that influence the health of future generations. This represents a critical gap in our understanding, particularly given that architectural exposures combine the chronic temporal characteristics, population-wide scope, and physiological activation patterns most strongly associated with epigenetic programming in environmental research. The theoretical framework of BEME emerges from this convergence of evidence, suggesting that built environments may function as persistent environmental modulators capable of creating lasting biological changes that extend across generations.

## 3. Architecturally Mediated Allostatic Overload

### Stress Response Systems and Built Environment Triggers

The physiological stress response to environmental threats is mediated through the coordinated activation of the hypothalamic–pituitary–adrenal (HPA) axis and the sympathetic-adreno-medullary (SAM) system, which work in concert to maintain homeostasis under challenging conditions [57]. Emerging evidence suggests that specific architectural features can function as environmental stressors, engaging these same neurophysiological pathways that typically respond to chemical or psychosocial threats [57].

Empirical substantiation for this relationship has been demonstrated in controlled experimental investigations examining the effects of spatial enclosure on stress physiology [58]. Fich et al. [58] employed a virtual-reality adaptation of the Trier Social Stress Test, demonstrating that participants exposed to fully enclosed room conditions exhibited a 73% elevation in salivary cortisol levels relative to those situated in open rooms with large apertures. Within the findings of Fich et al. [58], of particular significance was the finding that the enclosed condition cohort also displayed markedly attenuated physiological recovery, indicative of prolonged hypothalamic–pituitary–adrenal (HPA) axis activation in response to spatial confinement.

Neuroimaging research has further served to elucidate the cerebral mechanisms underlying architectural stress responses. Employing functional magnetic resonance imaging (fMRI), Vartanian et al. [59] determined that low ceiling heights and enclosed spatial conditions selectively engaged the anterior midcingulate cortex, a region critically implicated in fear processing and autonomic nervous system regulation. This pattern of neural activation suggests that spatially constrained proportions precipitate sympathetic–adrenal–medullary (SAM) axis responses analogous to those characteristically observed during threat detection paradigms. Emerging evidence suggests that the amygdala may also explain the built environment modulation of emotional reactivity and stress. fMRI studies have shown that even a brief one-hour walk in a natural setting compared to an urban environment can reduce amygdala activation [60]. Complementary structural MRI analyses reveal a positive association between proximity to forest cover and markers of amygdala integrity, such as cell density and myelin content, suggesting that vegetative environments may help to protect the amygdala’s structural resilience over time [61].

Taken together, these findings support a model in which the built environment and landscape exposure may modulate brain function and structure, affecting emotional regulation and vulnerability to stress. Beyond spatial characteristics, visual elements of the built environment also influence stress physiology. Architectural façades featuring high-contrast, repetitive patterns that deviate from natural statistical properties have been shown to provoke measurable autonomic responses, including increased skin conductance and elevated self-reported discomfort levels, reflecting heightened sympathetic nervous system arousal [62]. Additionally, the temporal aspects of environmental exposure play a crucial role in stress system activation. Exposure to artificial lighting during evening hours has been demonstrated to suppress nocturnal melatonin synthesis, effectively shortening the natural dark period (scotoperiod) and disrupting circadian regulation [63]. This circadian disruption subsequently elevates cortisol secretion and increases blood pressure, creating a cascade of physiological dysregulation [64]. Collectively, these findings establish that specific features of the built environment can function as persistent environmental stressors, activating the same HPA and SAM pathways that mediate epigenetic programming in response to other forms of environmental challenge.

A critical distinction between built environment stressors and other environmental challenges lies in their temporal characteristics. While chemical exposures or psychosocial stressors may be intermittent or acute, built environment features represent a near-constant element of daily human experience. This continuous exposure to stress-inducing spatial configurations, visually discordant stimuli, and circadian-disrupting lighting creates conditions conducive to allostatic overload, a state characterised by sustained activation of stress response systems and chronic elevation of cortisol and catecholamine levels [65].

Emerging lines of evidence suggest a plausible hypothesis whereby the sustained nature of exposure to built environment stressors, through cumulative physiological strain, could create conditions conducive to longer-term modulation of gene regulation. Under this proposed framework, epigenetic mechanisms may represent one pathway through which built environment stress exposure contributes to altered vulnerability across neuropsychiatric, immune, and metabolic domains.

## 4. The Built Environment-Modulated Epigenetics Framework

### 4.1. Theoretical Integration and Proposed Pathway

**Figure 1:**
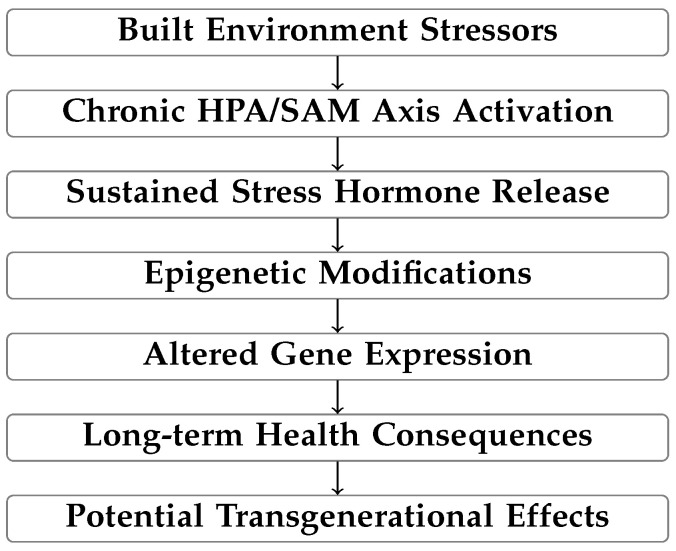
Proposed pathway underlying the built environment-modulated epigenetics (BEME) hypothesis. Chronic exposure to built environment stressors is hypothesized to contribute to sustained HPA/SAM axis activation and stress hormone release, leading to epigenetic modifications, altered gene expression, long-term health consequences, and potential transgenerational effects.

The built environment-modulated epigenetics (BEME) hypothesis has been developed based on the integration of well-established findings in environmental epigenetics and neuroarchitecture. This paper proposes the following theoretical pathway linking exposure to built environment stressors to epigenetic modifications (illustrated in Figure 1 below):

This pathway extends the framework of architecturally mediated allostatic overload by proposing that the health consequences of chronic built environment stress exposure include not only physiological wear and tear but also lasting molecular changes that modify stress response systems and other critical biological pathways. The theoretical framework rests on the premise that the built environment, through its capacity to induce chronic stress responses [66,67], may engage the same epigenetic machinery that has been extensively documented in response to a range of environmental stressors such as chemical toxins, air pollution, and psychosocial stressors [5,68].

The theoretical basis of this potential pathway is supported by several converging lines of evidence (see Section 2 above). First, mechanistic precedent exists that demonstrates that environmental stressors that activate the HPA axis can create epigenetic modifications [4]. This activation pathway is similar to the same physiological pathways that built environment stressors can activate [7]. The glucocorticoid and catecholamine cascades triggered by spatial confinement, visual discord, or circadian disruption may be the same stress response mechanism pathways as those documented in environmental epigenetics research [69,70]. Second, the chronic, sustained nature of built environment exposure potentially creates the prolonged activation conditions that environmental epigenetics studies have identified as most conducive to stable DNA methylation and histone modifications [71]. Third, target gene overlap exists between the stress-responsive genes most affected by environmental epigenetic programming and those likely to be affected by chronic exposure to built environment stressors, particularly genes associated with the HPA axis function, inflammatory responses, and circadian rhythms [72].

As summarised in Table 1, a range of built environment exposures may contribute to distinct stress-response pathways and associated epigenetic modifications within the proposed BEME framework.

### 4.2. Built Environment-Modulated Epigenetics (BEME) Biomarkers

To advance the field of BEME, we argue that testing the proposed framework will require the identification and articulation of a candidate set of physiological and molecular biomarkers capable of capturing the built environment stress related epigenetic modulation. It is proposed that this standardised panel could serve as a foundational tool for empirical research, enabling consistent measurement and comparison across studies. This canonical set could include, but is not limited to, the following categories and examples:

- Endocrine Indicators: Measures like cortisol levels to gauge stress responses and autonomic nervous system modulation influenced by architectural design elements [78]. These markers would be deliberately selected for their potential to identify subtle, built environment-induced epigenetic changes. Endocrine indicators can be utilised to help identify environmental stressors in the built environment before exploring their epigenetic consequences.
- Inflammatory Indicators: Cytokines such as interleukin-6 (IL-6) [73,74].
- Epigenetic Indicators: Gene-specific markers like methylation patterns and brain-derived neurotrophic factor (BDNF) [77], alongside broader metrics like epigenetic ageing clocks [79], to detect heritable modifications linked to chronic built environment stressors.

These biomarkers are envisioned primarily for research validation purposes, allowing investigators to test the validity of the BEME framework, quantify effect–response relationships, and refine theoretical models through longitudinal studies. Importantly, at this nascent stage, these indicators are not intended for clinical diagnostic or therapeutic applications; their role is to build a robust evidence base, ensuring that future translations to practice are grounded in rigorous science rather than premature assumptions. By establishing this canonical set, the field can foster interdisciplinary collaboration, standardise methodologies, and accelerate findings at the intersection of built environment design and epigenetics.

The BEME framework generates several critical research priorities that first need to be addressed to validate and refine the theoretical framework. The absence of direct empirical evidence for built environment-modulated epigenetic effects represents the most significant limitation of the current framework, necessitating systematic investigation across multiple research domains and methodological approaches. Direct testing of BEME effects constitutes the most urgent empirical need for validating the theoretical framework. These foundational studies should employ both controlled laboratory investigations and real-world observational research to establish whether architectural stress exposure creates measurable epigenetic modifications [80]. Animal model studies offer particular advantages for initial validation, as they permit controlled manipulation of built environment variables while eliminating many of the confounding factors that complicate human research. Rodent studies examining epigenetic responses to spatial confinement, visual pattern exposure, and lighting manipulation could provide initial proof-of-concept evidence for architectural epigenetic programming [81]. This is widely supported by the decades-long research done on rodents that has informed our understanding of environmental enrichment but not yet of stressors [76,82]. There also exists the need for more studies that measure DNA methylation and histone modifications in stress-responsive brain regions, including the hippocampus, prefrontal cortex, and hypothalamus, following chronic exposure to specific built environment stressors [83]. In parallel to the above studies, human studies are needed that examine epigenetic modifications in populations with well-defined built environment exposure characteristics, utilising peripheral biomarkers such as blood or saliva to assess systemic epigenetic changes in response to built environment stress exposure.

Longitudinal studies tracking individuals from initial built environment exposure through epigenetic modifications to subsequent health outcomes represent essential research for establishing causal relationships and identifying critical exposure periods. Prospective cohort studies beginning during critical developmental periods, such as prenatal maternal exposure or early childhood environmental programming [84], may provide the strongest evidence for causal relationships between built environment characteristics and health outcomes mediated through epigenetic mechanisms. Such studies offer the opportunity to integrate detailed and targeted built environment characteristic exposure assessments with repeated epigenetic biomarker collection and comprehensive health outcome monitoring to establish temporal relationships between environmental exposure, molecular programming, and disease development.

Transgenerational studies examining whether BEME can be transmitted represent perhaps an essential component of empirical validation. Animal model research may provide initial evidence for transgenerational BEME effects [85], as controlled breeding studies can isolate environmental exposures while tracking epigenetic and phenotypic changes across generations. Additionally, intervention studies, testing whether modifications to built environments can reduce or prevent adverse epigenetic changes, may provide a valuable avenue of research. Such studies offer the potential to examine both reduction in maladaptive epigenetic programming through improved built environment design and the mediation of existing epigenetic modifications through targeted built environment remediation. Randomised controlled trials of specific built environment characteristics, such as lighting interventions, spatial modifications, or biophilic design elements, may provide more definitive evidence for causal relationships between specific built environment design elements and epigenetic changes.

### 4.3. Methodological Considerations and Challenges

BEME is at present a theoretical model. The translation of BEME from a theoretical hypothesis to an empirically validated model faces substantial methodological challenges that must be systematically addressed to generate valid and reliable empirical evidence. These challenges span exposure assessment, confounding variables, temporal dynamics, and individual-level variation considerations that are fundamental to environmental epigenetics research but particularly complex in built environment contexts.

Confounding variables represent perhaps the most significant methodological challenge in BEME research, as built environment exposures are often inextricably linked with socioeconomic factors, lifestyle characteristics, and other environmental exposures that independently influence epigenetic programming. Individuals living in stress-inducing built environments often experience concurrent exposure to air pollution, noise, social stressors, and economic disadvantage that may create epigenetic modifications independent of the specific built environment features under examination. Careful study design approaches are necessary to isolate architectural effects from these confounding influences.

Exposure measurement represents another fundamental methodological challenge that requires development of novel assessment approaches specifically designed for chronic built environment stress evaluation. Unlike discrete chemical exposures or acute psychosocial stressors that can be measured through conventional environmental monitoring or psychological assessment instruments [75,86,87], built environment stress exposure operates through complex, multidimensional environmental characteristics that may interact in non-linear ways to influence physiological responses. Valid architectural stress exposure assessment may require integration of objective environmental measurements (lighting conditions, spatial dimensions, visual characteristics, acoustic properties) with subjective experience assessments (perceived stress, environmental satisfaction, behavioural responses) and physiological monitoring (cortisol patterns, autonomic nervous system activity, sleep quality indicators), in parallel with animal studies. Emerging technologies, such as IoT, wearable sensors, environmental monitoring systems, and smartphone-based ecological monitoring assessment tools, may enable more comprehensive and ecologically valid measurement of built environment stress exposure in real-world settings.

Epigenetic stability considerations complicate study design and interpretation in BEME research, as the temporal dynamics of environmentally induced epigenetic changes remain incompletely understood. A potential challenge for BEME is the need to account for both acute epigenetic responses that may occur rapidly following environmental exposure and chronic epigenetic programming that develops over extended periods of built environment stress exposure. Additionally, the potential for epigenetic restoration following built environmental design improvement requires consideration in study design, as intervention studies may need to account for varying recovery timescales depending on the specific epigenetic mechanisms involved.

Individual variation in susceptibility to both architectural stress and epigenetic programming represents a critical methodological consideration that may create substantial heterogeneity in research findings. Genetic polymorphisms affecting stress sensitivity, including variations in glucocorticoid receptor function, stress-responsive neurotransmitter systems, and DNA methylation machinery, may moderate individual responses to built environment stressors. Additionally, previous environmental exposures, developmental history, and current health status may influence epigenetic responsivity to built environment features.

### 4.4. Theoretical Limitations

The BEME framework, while theoretically grounded in established principles from epigenetics and neuroarchitecture research, remains at this stage a theoretical hypothesis. These limitations span both the theoretical construction of the framework and the practical challenges associated with validating and implementing BEME concepts.

Empirical validation represents the most significant limitation of the BEME framework, as the theory remains untested through direct experimental investigation. The framework requires extensive empirical validation across multiple levels, from molecular epigenetic modifications and physiological stress responses to long-term health outcomes, before the theory can be considered scientifically established. The absence of direct evidence for BEME effects means that the framework currently represents a research hypothesis rather than an established scientific model. Closing this empirical gap requires a phased research agenda: first, proof-of-concept studies showing that built environment stressors can produce measurable epigenetic changes; second, work mapping exposure–response relationships over time; and third, studies linking these changes to health outcomes.

Mechanistic specificity represents another fundamental limitation, as the theoretical framework relies on extrapolation from established environmental epigenetics mechanisms rather than direct investigation of built environment stress-specific pathways. While the general mechanisms of environmental epigenetic programming through stress hormone-mediated DNA methylation and histone modifications are well-established, the specific molecular pathways through which built environment stressors might induce epigenetic modifications require detailed mechanistic investigation. Understanding these mechanistic distinctions is essential for developing targeted interventions and developing an understanding of which built environment features are most likely to create potential epigenetic consequences.

## 5. Conclusions

The concept of BEME represents a theoretical extension of established frameworks in environmental epigenetics and neuroarchitecture, proposing that the health impacts of stress-inducing built environments may include lasting epigenetic modifications with profound implications for individual and population-level health across generations. This framework emerges from the integration of four well-established research domains: environmental stress-induced epigenetic programming, architecturally mediated stress responses, transgenerational epigenetic inheritance, and environmental enrichment. While the framework remains hypothetical and requires extensive empirical validation, it addresses critical gaps in our understanding of how built environments influence human health and potentially offers new opportunities for health promotion through design.

The implications of BEME are far-reaching, suggesting that built environments may function as biological and epigenetic modulators that create biological legacies extending across generations. This perspective fundamentally challenges traditional approaches to architectural design and urban planning, positioning these disciplines as critical determinants of long-term biological modulation and population health. The built environment is, arguably, among the most pervasive and modifiable environmental contexts shaping daily human experience. Exploring its potential role in epigenetic regulation may offer a useful lens for understanding how chronic environmental conditions contribute to long-term health trajectories and population-level disparities, and the mechanisms through which environmental enrichment might counteract the maladaptive epigenetic changes by promoting stress resilience. While the BEME framework remains theoretical, it highlights the importance of investigating whether built environment design-related stress exposures have biological consequences that extend beyond immediate physiological responses. Clarifying these relationships through empirical research could inform more evidence-aware approaches to architectural and urban design, and have profound health ramifications for current and future generations.

## Figures and Tables

**Table 1:** Summary of principal built environment exposure categories and their corresponding (documented or hypothesised) DNA methylation and histone modification patterns, with associated downstream stress-responsive genes and outcomes within the built environment-modulated epigenetics (BEME) framework.

Built Environment Exposure Category	Hypothesised Stress Pathway	Hypothesised DNA Methylation Pattern	Hypothesised Histone Modification Pattern	Hypothesised Associated Downstream Genes/Outcomes
Spatial enclosure & low ceiling height	Sustained HPA axis activation; anterior midcingulate engagement; elevated salivary cortisol with attenuated recovery [58,59].	Hypothesised hypermethylation of glucocorticoid receptor regulatory regions, consistent with patterns reported under chronic glucocorticoid exposure [4,6].	Predicted reduction in activating histone acetylation (e.g., H3K9ac, H3K27ac) at stress-responsive promoters under chronic HPA activation [18].	*NR3C1*, *FKBP5*—altered HPA reactivity, anxiety-related phenotypes [4,51].
Visually discordant facades & high-contrast repetitive patterns	SAM axis arousal; increased skin conductance and self-reported discomfort indicating sympathetic engagement [62].	Hypothesised differential methylation of catecholaminergic and inflammatory loci under repeated sympathetic activation, paralleling psychosocial-stress findings [36].	Predicted shifts in H3K4me3/H3K27me3 balance at inflammation- and arousal-related promoters under chronic SAM signalling [27].	*TH*, *IL-6*—sympathetic tone, low-grade inflammation [73,74].
Artificial/evening light exposure & circadian disruption	Suppressed nocturnal melatonin, shortened scotoperiod, elevated cortisol, raised blood pressure [63,64].	Documented methylation changes at clock and inflammation-related genes following circadian disruption and shift-work-type exposures [6].	Disrupted rhythmic histone acetylation at circadian-controlled loci, altering temporal gene expression programmes [26].	*BMAL1*, *PER*, *CRY*—circadian regulation, metabolic and cardiovascular risk [64].
Urban density, neighbourhood disadvantage & adverse riskscapes	Cumulative chronic stress, co-exposure to noise and air pollution, sustained allostatic load [50,52].	Empirically observed differential methylation across stress, inflammation and cardiovascular networks linked to neighbourhood disadvantage [50,53].	Altered chromatin accessibility at inflammatory promoters consistent with chronic psychosocial-stress signatures [36].	*NR3C1*, *FKBP5*, *IL-6*—depression, PTSD and cardiovascular risk [50,53].
Air pollution & particulate exposure (built environment coupled)	Oxidative stress, systemic inflammation, accelerated biological ageing [35,75].	Empirically documented PM2.5-associated methylation shifts and accelerated epigenetic-clock measures [34,35].	Altered acetylation and methylation marks at oxidative stress and inflammation loci [33].	Inflammation and oxidative stress pathways; epigenetic-age acceleration markers [34].
Greenspace access & biophilic design (protective exposure)	Reduced amygdala activation, lower cortisol, parasympathetic engagement [60,61].	Empirically observed increased placental methylation of the serotonin receptor gene with greater residential greenness [56]; slower epigenetic ageing with sustained nature exposure [55].	Increased BDNF-associated histone acetylation under enriched environments, supporting transcription and neuroplasticity [44,76].	*HTR2A*, *BDNF*—neurodevelopment, neuroplasticity, stress resilience [47,56].
Environmental affordances for physical activity (protective exposure)	BDNF release through MET-reaching activity; hippocampal neurogenesis and stress recovery [47,49].	Hypothesised reduction in stress-induced hypermethylation of neurotrophic and HPA-regulatory genes through repeated activity-induced signalling [77].	Increased histone acetylation at the BDNF locus, enhancing transcriptional activity [44,76].	*BDNF*, *NR3C1*—neuroplasticity, learning, mood regulation, stress resilience [47,77].

Note. Where empirical evidence directly links a built environment exposure to an epigenetic outcome (e.g., green space–HTR2A methylation; PM2.5–epigenetic ageing; neighbourhood disadvantage–cardiovascular methylation), entries are reported as documented findings. Where evidence is currently indirect, entries are presented as theoretically grounded hypotheses extrapolated from convergent findings in environmental epigenetics, neuroarchitecture, and stress biology, consistent with the theoretical scope of the BEME framework outlined in Section 2, Section 3 and Section 4. HPA = hypothalamic–pituitary–adrenal; SAM = sympathetic-adreno-medullary.

## Data Availability

No new data were created or analysed in this study. Data sharing is not applicable to this article.
